# Self-Reported Physical Activity and Relations to Growth and Neurotrophic Factors in Diabetes Mellitus: The Framingham Offspring Study

**DOI:** 10.1155/2019/2718465

**Published:** 2019-01-09

**Authors:** Nicole L. Spartano, Kendra L. Davis-Plourde, Jayandra J. Himali, Joanne M. Murabito, Ramachandran S. Vasan, Alexa S. Beiser, Sudha Seshadri

**Affiliations:** ^1^Department of Endocrinology, Diabetes, Nutrition & Weight Management, Boston University School of Medicine (BUSM), Boston, MA, USA; ^2^Framingham Heart Study, Framingham, MA, USA; ^3^Department of Biostatistics, Boston University School of Public Health (BUSPH), Boston, MA, USA; ^4^Department of Neurology, BUSM, Boston, MA, USA; ^5^Departments of Medicine and Epidemiology, BUSM and BUSPH, Boston, MA, USA; ^6^Glenn Biggs Institute for Alzheimer's and Neurodegenerative Diseases, University of Texas Health Sciences Center, San Antonio, TX, USA

## Abstract

**Aims:**

Circulating insulin-like growth factor- (IGF-) 1, vascular endothelial growth factor (VEGF), and brain-derived neurotrophic factor (BDNF) levels are often lower in individuals with diabetes mellitus (DM) and are important for repairing vascular and neuronal dysfunction. The purpose of this investigation was to determine the cross-sectional relations of physical activity to circulating concentrations of IGF-1, VEGF, and BDNF in individuals with and without DM.

**Methods:**

In 1730 participants from the Framingham Offspring Study examination cycle 7, including those with DM (*n* = 179, mean age 64 years, 39% women) and without DM (*n* = 1551, mean age 60 years, 46% women), we related self-reported physical activity variables to circulating concentrations of IGF-1, VEGF, and BDNF using linear multivariable regression models. We also tested for interactions by age. Participants with prevalent cardiovascular disease, stroke, and dementia or taking hormone replacement therapy were excluded.

**Results:**

In participants with DM, more ambulatory physical activity was associated with higher IGF-1 levels (*β* ± standard error (SE) = 0.22 ± 0.08, *p* = 0.009), and more total physical activity was related to higher BDNF levels (*β* ± SE = 0.18 ± 0.08, *p* = 0.035), but physical activity was not significantly related to circulating VEGF. In participants without DM, no associations were observed. Moreover, in the examination of interactions by age, the association of ambulatory physical activity with IGF-1 levels was only observed in older adults with DM (age ≥ 60 years, *β* ± SE = 0.23 ± 0.11, *p* = 0.042) but not in middle-aged adults with DM (age < 60 years, *β* ± SE = 0.06 ± 0.13, *p* = 0.645).

**Conclusion:**

Our results suggest that more physical activity is associated with higher circulating IGF-1 and BDNF in participants with DM. These results, dissecting interactions by both age and DM status, may also help to explain some of the inconsistent results in studies relating physical activity to growth and neurotrophic factors.

## 1. Introduction

Individuals with diabetes mellitus (DM) have a higher burden of vascular and neurological damage than healthy individuals, potentially explaining increased rates of cardiovascular disease and cognitive decline in DM patients [1–4]. Physical activity is associated with the prevention and better management of DM complications [5]. Increasing physical activity through exercise training programs has also been shown to improve vascular, peripheral nerve, and cognitive function through vascular remodeling, angiogenesis, and neurogenesis [6–10]. Therefore, exercise interventions may be especially important for the prevention of complications of DM.

Growth factors with angiogenic and neurotrophic properties (such as insulin-like growth factor- (IGF-) 1, vascular endothelial growth factor (VEGF), and brain-derived neurotrophic factor (BDNF)) are implicated in vascular and neurological repair in both animal and human studies [11–13]. Animal models suggest that an imbalance or impaired responsiveness of these factors may partially explain higher vascular burden and neuropathy in old age and DM [14, 15]. Moreover, human exercise intervention studies have demonstrated an increase in circulating levels of IGF-1, VEGF, and BDNF [7, 16–18], although more consistently in older adults [7, 19] compared to younger populations [8, 19–21]. It will be important to understand whether the association of exercise and physical activity with growth and neurotrophic factors may be higher in other populations with high vascular or neurological dysfunction, such as in persons with DM.

In the present investigation, we examined the associations of physical activity with circulating concentrations of IGF-1, VEGF, and BDNF in a community-based sample and explored these relations in subsets of our study population, such as individuals with and without DM. We hypothesized that greater physical activity would be associated with higher circulating IGF-1, VEGF, and BDNF levels especially in older adults and those with DM. We also hypothesized that ambulatory activity is positively associated with circulating levels of growth and neurotrophic factors because previous studies have shown the importance of walking in the prevention of DM complications [6, 22].

## 2. Materials and Methods

**Figure 1:**
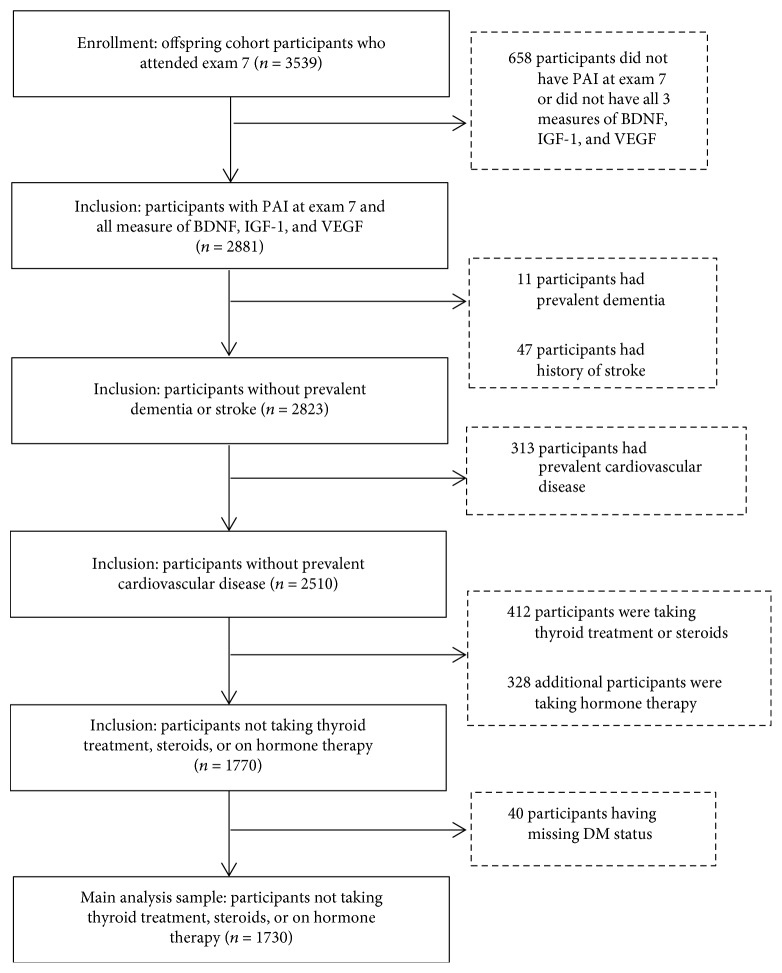
Diagram illustrating derivation of analytic sample used for the main analysis. Abbreviations: DM: diabetes mellitus; PAI: physical activity index; IGF-1: insulin-like growth factor-1; VEGF: vascular endothelial growth factor; BDNF: brain-derived neurotrophic factor.

The Framingham Offspring Study is a prospective cohort study that began recruitment in 1971, in Framingham, Massachusetts [23]. Participants have been assessed once every four to eight years. These participants are the offspring and offspring's spouses of the Original Framingham Heart Study cohort. Framingham Offspring Study participants who attended examination cycle 7 (1998-2001, *n* = 3539, Figure 1) were included if they had completed physical activity questionnaires and had blood IGF-1, VEGF, and BDNF measurements at cycle 7 (*n* = 2881). Participants with prevalent cardiovascular disease, stroke, or dementia, conditions that could affect physical activity or the accuracy of self-reported physical activity assessments (*n* = 371), were excluded. Others were excluded for being on estrogen replacement therapy, steroids, or thyroid hormones at cycle 7 (*n* = 740) because these treatments could impact circulating growth factor levels. DM status was defined as a fasting glucose of ≥126 mg/dl or self-reported use of insulin or oral hypoglycemic agents at cycle 7. Persons whose DM status was uncertain were also excluded (*n* = 40). The final study sample included 1730 participants. All participants provided written informed consent. The study protocols were approved by the institutional review board at Boston University Medical Center.

### 2.1. Laboratory Measurements of Growth and Neurotrophic Factors

Blood samples were collected in 1998-2001 from fasting participants during examination cycle 7 and stored frozen at −80°C. Serum BDNF (pg/ml), VEGF (pg/ml), and IGF-1 (ng/ml) concentrations were measured using commercial ELISA assays (R&D Systems, Minneapolis, MN) in 2010-2011. In our laboratory's quality control tests, intra- and interassay coefficients of variation (CVs) for BDNF were 4.8 and 7.6%, for VEGF were 3.9 and 8.4%, and for IGF-1 were 3.4 and 4.5%, respectively [24, 25].

### 2.2. Physical Activity Questionnaires

The physical activity index (PAI) was a composite score of total physical activity, constructed for each subject by weighting each hour in a typical day based on their activity level. The PAI has previously been used in predicting risk of cardiovascular disease mortality and dementia in the Framingham Heart Study [26, 27]. Participants were asked to report the number of hours in a typical day spent sleeping (weighting factor (WF) = 1) and in sedentary (WF = 1.1), slight (WF = 1.5), moderate (WF = 2.4), and heavy activities (WF = 5) [26]. For example, a completely sedentary person could receive a PAI score as low as 26 and a laborer involved in heavy physical activity could receive a score of 42. We also used the number of hours reported as sedentary time as a separate variable. Finally, participants were asked to report the number of flights of stairs and city blocks walked each day, which were added together to quantify the total amount of ambulatory (walking) physical activity performed, because walking is the most common form of physical activity performed by older adults.

### 2.3. Statistical Analysis

In order to normalize the distributions of the physical activity measures, a negative inverse transformation was used for PAI, a square root transformation for sedentary time, and a natural logarithmic transformation for the ambulatory activity reported. We standardized each of these independent variables to a mean of 0 and a standard deviation (SD) of 1 to facilitate comparison across various physical activity variables. In addition, VEGF was natural log transformed because of its skewed distribution. All outcome variables were also standardized to a mean of 0 and a SD of 1.

We used multivariable linear regression Model 1, adjusting for age and sex, to assess associations between each of the three physical activity predictors (total physical activity [PAI], sedentary time, and total ambulatory physical activity) and each of IGF-1, VEGF, and BDNF (dependent variables). In Model 2, we additionally adjusted for body mass index (BMI, which we natural log transformed to normalize the skewed distribution), current smoking, total cholesterol, triglycerides, lipid medication, and APOE-*ε*4 status (ε4− vs. ε4+). We observed significant interactions by DM status, which further justified our *a priori* decision to stratify all analyses by DM status (interaction *p* = 0.024 for sedentary time to VEGF; interaction *p* = 0.076 for total physical activity to BDNF; and interaction *p* = 0.081 for ambulatory activity to IGF-1; Supplemental Table 1). Because we observed a strong relation between age and DM, we also tested whether interactions with DM were present independent of age. Furthermore, we performed sensitivity analysis without excluding individuals taking hormone replacement therapy to assess the possibility that we are introducing more bias by excluding this set of older women. Significance was set at *p* < 0.10 for tests of interactions and *p* < 0.05 for all other tests. All analyses were performed using SAS version 9.4 (SAS Institute Inc., Cary, NC).

## 3. Results

Framingham Offspring Study participants without DM (*n* = 1551) self-reported climbing a median of 6 flights of stairs, walking 12 blocks, and sitting for a median of 6 hours per day (Table 1). Participants with DM (types 1 and 2, *n* = 179) reported similar total physical activity but reported climbing fewer flights of stairs (*p* = 0.031, adjusted for age) and walking a median of only 6 blocks, just half as many blocks as reported by participants without DM (*p* < 0.0001, adjusted for age). After adjusting for age, time spent in sedentary activities also differed between people with DM and without DM (*p* = 0.027).

Circulating IGF-1 levels were significantly lower in participants with DM compared to those without DM, after adjusting for age (*p* = 0.014, Table 2), but there were no significant differences in serum VEGF or BDNF by DM status. In participants with DM, more ambulatory activity was associated with having higher blood IGF-1 levels (*β* ± standard error (SE) = 0.22 ± 0.08, *p* = 0.009; Table 3, Model 2), after adjusting for age, sex, BMI, smoking, total cholesterol, triglycerides, lipid medication, and APOE4 genotype. Walking the equivalent of 50% more city blocks or flights of stairs was associated with having 0.1 standard deviation unit higher IGF-1 concentration. In the same subset of participants with DM, total physical activity was associated with higher serum BDNF concentrations (*β* ± SE = 0.18 ± 0.08, *p* = 0.035; Table 3). In participants without DM, we observed no significant associations of physical activity with either serum IGF-1 or BDNF (Table 4). No associations were observed between physical activity and circulating VEGF in participants with or without DM.

In order to determine whether the observed associations of physical activity measures with growth and neurotrophic factors in DM participants were due to their older ages, we performed a secondary analysis. We identified that, in the subgroup of participants < 60 years, the interactions by DM remained statistically significant for the relation of total physical activity to serum BDNF (interaction *p* = 0.077, Supplemental Table 1). Conversely, in participants ≥ 60 years, the interaction by DM was significant for the relation of both total and ambulatory physical activity to serum IGF-1 levels (interaction *p* = 0.032 and *p* = 0.025, respectively). For the relation of total physical activity to circulating BDNF concentrations in participants with DM (Table 3), there did not appear to be any differences in the significance of the association by age strata (Supplemental Table 2). However, for the relation of ambulatory activity to serum IGF-1 levels in DM (Table 3), the size of the association was stronger and only significant for participants with DM ≥ 60 years (*β* ± SE = 0.23 ± 0.11, *p* = 0.042, Supplemental Table 2). Therefore, it does appear that both age and DM status influenced the relation of physical activity to serum IGF-1 levels, although three-way interactions were not tested.

To further elucidate the interactions by age and DM status, we reported that older age was associated with lower IGF-1 concentrations (only significant in participants without DM, *p* < 0.0001, Supplemental Table 3). One possible explanation of our results is that the association of ambulatory activity with IGF-1 only in those ≥60 years may be due to both age and DM status driving down IGF-1 (as presented in Table 2 and Supplemental Table 3), allowing for a more pronounced effect of physical activity on IGF-1.

Finally, in order to confirm the appropriateness of excluding women receiving hormone replacement therapy, we performed sensitivity analysis where we removed this exclusion (*n* = 2098) and observed similar results (data not shown).

## 4. Discussion

In our Framingham Offspring Study, in a community setting, our findings were threefold: firstly, in persons with DM, we observed that greater physical activity was associated with higher circulating IGF-1 and BDNF levels; secondly, the association with IGF-1 was only significant in older adults (≥60 years) with DM; and finally, no significant relations were observed in adults without DM. Previous studies have reported inconsistent associations of physical activity with levels of growth and neurotrophic factors [7, 8, 16–21], with the most consistent effects in animal and human studies suggesting elevations in levels of IGF1 and BDNF on walking or running. But there have been limited efforts to distinguish among these relations in different subsets of the population, such as individuals with DM and by age categories.

Studies revealing positive relations of physical activity to growth and neurotrophic factors have typically been in older adults [19, 21]. During later decades, aging of the vascular and neurological systems leads to a greater risk for cardiovascular disease and cognitive impairment as well as peripheral vascular disease and peripheral neuropathy [28, 29]. IGF-1, VEGF, and BDNF have been termed *angioneurins* [30], influencing both the vascular and neurological domains [11–13]. Thus, the relation of physical activity to these factors may have specific implications for preventing complications in older adults and for individuals with diseases, such as DM, that also cause vascular and neurological dysfunction.

In the United States, it has been reported that individuals with DM are less active than individuals without this disease [5]. Inactivity may be particularly harmful in individuals with DM because exercise programs are one of the major lifestyle interventions available to reduce DM complications [6, 9]. It is unclear whether growth and neurotrophic factors modulate the beneficial effect of physical activity in individuals with DM, but we present evidence to suggest that physical activity was more strongly related to circulating IGF-1 and BDNF levels in individuals with DM, even after adjusting for covariates and potential confounders.

### 4.1. Physical Activity, Growth/Neurotrophic Factors, and Angiogenesis/Neurogenesis

One mechanism by which physical activity may improve vascular and neurological systems, especially in older individuals or those with DM, is through the impact of growth and neurotrophic factors on angiogenesis and neurogenesis. Evidence from cell culture experiments suggests that much of the angiogenic effect of exercise is dependent on VEGF [31], although we did not observe associations of self-reported physical activity with VEGF levels. Moreover, IGF-1 indirectly stimulates both angiogenesis and neurogenesis through the induction of muscle hypertrophy [32, 33]. The larger tissues require more rapid oxygen and nutrient supply and innervation, so there are extensions of capillary and nerve networks necessary for function.

The neurogenic effect of physical activity may also be due to increased BDNF levels. In rats, acute exercise increases proBDNF and cleaves and activates mature BDNF in the brain, promoting synthesis of synapsis I and other factors, involved in axonal elongation, maintenance of synaptic contacts, and the release of neurotransmitters [34]. Although acute exercise increases circulating BDNF, chronic exercise programs have not reliably demonstrated an increase in BDNF [8, 35, 36]. It is possible that these negative results are due to confounding factors that remain unaccounted for. Therefore, large observational studies that effectively adjust for confounding may be especially important. Additionally, between-study differences in study population characteristics, exercise intensities, and methods of BDNF measurement (plasma vs. serum, the various BDNF isoforms measured by the different assays) may also influence results.

The angiogenic and neurogenic properties of growth and neurotrophic factors may be especially important in older age and in DM. One debilitating complication of DM involves peripheral neuropathy, a painful condition that impacts mobility and motor function [6]. Glycemic control, initiated through exercise, weight loss, or medication, is often the only treatment offered to reduce this pain and prevent progression of the disorder [6, 37]. Exercise can have a positive impact on peripheral neuropathy, decreasing symptoms or even decreasing the risk of developing this disease [6].

In our Framingham Offspring Study, we observed a significant relation of total physical activity to higher BDNF levels in participants with DM. Our observations are relevant because BDNF may play a role in the prevention of DM complications through maintaining peripheral nerve regeneration or synaptic connectivity [34]. Previous studies have also reported a stronger effect of the relation of physical activity to BDNF in older age [7], although we did not observe this relation in our study. One potential reason for these discrepant results may be that in our sample, BDNF levels were not significantly different in older participants compared to younger participants, perhaps because the Framingham Offspring Study has a mean age in the 60s and only a small percentage of elderly participants. Previous studies have typically reported a decrease in circulating BDNF levels with age [38, 39], which may impact the variability of BDNF levels within a population and may partially explain neurological dysfunction in older age.

### 4.2. Physical Activity, Growth/Neurotrophic Factors, and Vascular Remodeling

Human exercise interventions result in vascular remodeling [10]. Exercise stimulates increased blood flow, which puts tension and shear stress on the arterial wall and initiates a cascade of growth and neurotrophic factor upregulation, in turn assisting in remodeling the arterial wall [40]. Mechanical forces on the vasculature also set off a growth hormone response cascade inducing secretion of IGF-1. Limited data from animal and cell culture experiments suggest that elevated IGF-1 and BDNF levels may also induce dilation of arteries via upregulation of elastin [41] and prostaglandin synthesis [42], respectively. However, IGF-1 is not strictly stimulated by hemodynamic forces. Despite greater arterial stiffness in older adults [29] or those with DM [43], an inverse association has been reported between circulating IGF-1 and arterial stiffness [44]. Moreover, circulating IGF-1 appears to be lower in older age and in individuals with DM, as previously demonstrated [45] and replicated in this investigation. These lower IGF-1 levels in older adults with DM may account for our results, possibly allowing for a greater effect of ambulatory physical activity on IGF-1 levels only in older adults with DM where IGF-1 levels are lower to begin with.

### 4.3. Physical Activity, Growth/Neurotrophic Factors, and Cerebral Plasticity

In addition to the role of growth and neurotrophic factors in the maintenance of vascular and nerve function at older ages and in DM, growth and neurotrophic factor stimulation may also be partially responsible for the cognitive benefit from physical activity observed in older adults [19] and possibly those with DM. There is evidence suggesting that IGF-1, VEGF, and BDNF can each cross the blood-brain barrier [17, 46–48], and in animal studies, when IGF-1, VEGF, and BDNF activities were experimentally suppressed or their access to the brain was blocked, the beneficial effect of exercise effect on neurogenesis was suppressed [46, 47, 49]. There have been promising human studies demonstrating the magnitude of exercise-induced increases in BDNF related to changes in hippocampal volume and brain connectivity in older adults [8, 19]. Our study provides supporting evidence for the role of growth and neurotrophic factors in physically active older adults with DM, which have possible implications for vascular remodeling, angiogenesis, and neurogenesis.

### 4.4. Strengths and Limitations

Due to the cross-sectional study design, we are unable to draw causal inferences or to detect transient influences of exercise on growth and neurotrophic factors. Another consideration is that our results rely on self-reported physical activity, known to introduce measurement error [50]. Thus, our results may actually underestimate the associations of physical activity with growth and neurotrophic factors. As true for any epidemiological study, blood samples were collected over a period of a few years. Thus, storage time of samples varied, potentially biasing growth and neurotrophic factor measurements. However, because storage times varied randomly, it would only serve to attenuate potential associations.

Observations in our Framingham Offspring Study sample were also predominantly on white individuals of European ancestry, limiting our ability to generalize results to other ethnicities. However, because of the large sample size of our main study, we were able to test for interactions by age and DM status among potential associations. We were also able to adjust for many sociodemographic variables that may be covariates or confounders in our statistical models, such as age, sex, BMI, smoking, total cholesterol, triglycerides, lipid medication, and APOE4 genotype. However, we acknowledge the limitation that there may be residual confounding by sociodemographic variables that cannot be measured with precision or were not included in our final models (such as diet, somatic diseases, or depressive symptoms). We were also unable to account for the potential influence of platelet counts, which are reported to correlate with BDNF levels [51]. Future studies should be designed to test the influence of these potential confounders.

One important confounder that we were able to consider was age, which is notable because many growth factors have been reported to decrease with age [20, 52]. The resulting low circulating growth/neurotrophic factors in older adults may provide an opportunity for physical activity to influence brain structure and function through the induction of growth or neurotrophic factors [7].

## 5. Conclusions

Our results suggest that physical activity is positively related to growth and neurotrophic factors, especially in subgroups of the population at risk for higher vascular and neurological dysfunction, such as in older adults and participants with DM. We also reported potential differences in the stimulation of growth and neurotrophic factors by different intensities of activity in subsets of the population. For example, in DM patients, ambulatory activity may be especially important for stimulating IGF-1, whereas light activity may play a role in stimulating BDNF. These data may be useful in a deeper understanding of how specific types of exercise interventions may differentially stimulate vascular remodeling, angiogenesis, neurogenesis, and cerebral plasticity to prevent or treat complications of DM.

## Figures and Tables

**Table 1:** Study characteristics of participants at Framingham Offspring examination cycle 7.

Demographic variables	Participants without DM *N* = 1551	Participants with DM *N* = 179	Age-adjusted *p*
Age, years, mean ± std (at examination 7)	60 ± 10	64 ± 8	<0.0001^∗^
Women, *n* (%)	712 (46)	69 (39)	0.044
BMI, kg/m^2^, median (Q1, Q3)	27 (25, 30)	31 (28, 36)	<0.0001
Current smoking, *n* (%)	191 (12)	18 (10)	0.911
Hypertension, *n* (%)	573 (37)	123 (69)	<0.0001
Triglycerides, mg/dl, mean ± std	127 ± 83	171 ± 100	<0.0001
Total cholesterol, mg/dl, mean ± std	201 ± 36	192 ± 37	0.0009
HDL cholesterol, mg/dl, mean ± std	53 ± 16	45 ± 14	<0.0001
Lipid med use, *n* (%)	215 (14)	60 (34)	<0.0001
Hypertension med use, *n* (%)	379 (24)	100 (56)	<0.0001
APOE *ε*4+, *n* (%)	310 (20)	41 (23)	0.342
Physical activity measures			
*Total physical activity* (PAI), median (Q1, Q3)	37 (33, 41)	37 (33, 42)	0.393
*Sedentary time*, hours/day, median (Q1, Q3)	6 (4, 8)	6 (4, 8)	0.027
Flights walked, median (Q1, Q3)	6 (3, 10)	5 (2, 10)	0.031
Blocks walked, median (Q1, Q3)	12 (3, 24)	6 (1, 12)	<0.0001
*Total ambulatory physical activity* Flights + blocks walked, median (Q1, Q3)	17 (9, 30)	12 (6, 22)	<0.0001

Abbreviations: DM: diabetes mellitus; BMI: body mass index; APOE: apolipoprotein E; HDL: high-density lipoprotein; PAI: physical activity index; Q: quartile. ^∗^This *p* value was not age-adjusted.

**Table 2:** Relations of DM status to circulating BDNF, IGF-1, and VEGF levels adjusted for age.

	Participants without DM *N* = 1551	Participants with DM *N* = 179	Age-adjusted *p*
*IGF-1, ng/ml *mean ± SE	120 ± 0.89	113 ± 2.64	**0.014**
*VEGF, pg/ml* median (Q1, Q3)	267 (159, 452)	276 (152, 428)	0.58
*BDNF, pg/ml *mean ± SE	23881 ± 210	24343 ± 623	0.48

Abbreviations: IGF-1: insulin-like growth factor-1; VEGF: vascular endothelial growth factor; BDNF: brain-derived neurotrophic factor; DM: diabetes mellitus. *p* values were calculated using analysis of covariance (ANCOVA). For variables not normally distributed, a transformation was applied to make the distribution more normal. Significant *p* values (*p* < 0.05) were set in bold for emphasis.

**Table 3:** The relations of physical activity variables to IGF-1, VEGF, and BDNF levels in participants with DM (*n* = 179).

Per SD of factor	Model	IGF-1		VEGF		BDNF	
		Beta ± SE	*p*	Beta ± SE	*p*	Beta ± SE	*p*
*Sedentary time*	1	−0.01 ± 0.08	0.853	0.14 ± 0.08	0.085	−0.04 ± 0.09	0.655
	2	0.03 ± 0.08	0.678	0.14 ± 0.08	0.088	−0.03 ± 0.09	0.747
*Total physical activity*	1	−0.05 ± 0.08	0.493	−0.01 ± 0.08	0.854	0.15 ± 0.08	0.061
	2	−0.07 ± 0.08	0.342	−0.003 ± 0.08	0.971	0.18 ± 0.08	**0.035**
*Ambulatory activity*	1	0.21 ± 0.08	**0.007**	−0.08 ± 0.08	0.333	−0.06 ± 0.09	0.510
	2	0.22 ± 0.08	**0.009**	−0.06 ± 0.09	0.522	−0.03 ± 0.10	0.736

Abbreviations: APOE4: apolipoprotein E4; IGF-1: insulin-like growth factor-1; VEGF: vascular endothelial growth factor; BDNF: brain-derived neurotrophic factors; SE: standard error; DM: diabetes mellitus. Model 1: adjusted for age and sex; Model 2: additionally adjusted for body mass index, smoking, total cholesterol, triglycerides, lipid medication, and APOE4. Ambulatory activity = number of flights of stairs and city blocks walked each day, which were added together to quantify the total amount of ambulatory (walking) physical activity. Ambulatory activity was natural log transformed, sedentary time was square root transformed, and total physical activity (PAI) was inverse transformed, but we reversed the sign for total physical activity to make higher values consistent with more physical activity. VEGF was also natural log transformed. All variables were standardized to a mean of 0 and SD of 1 to facilitate comparison. Significant *p* values (*p* < 0.05) were set in bold for emphasis.

**Table 4:** The relations of physical activity variables to IGF-1, VEGF, and BDNF levels in participants without DM (*n* = 1551).

Per SD of factor	Model	IGF-1		VEGF		BDNF	
		Beta ± SE	*p*	Beta ± SE	*p*	Beta ± SE	*p*
*Sedentary time*	1	0.02 ± 0.02	0.474	−0.04 ± 0.03	0.128	0.02 ± 0.02	0.539
	2	0.02 ± 0.02	0.341	−0.04 ± 0.03	0.088	0.02 ± 0.02	0.388
*Total physical activity*	1	0.01 ± 0.02	0.695	0.04 ± 0.03	0.101	0.006 ± 0.03	0.819
	2	0.01 ± 0.02	0.797	0.05 ± 0.03	0.082	0.002 ± 0.03	0.938
*Ambulatory activity*	1	0.04 ± 0.03	0.113	0.03 ± 0.03	0.311	−0.02 ± 0.03	0.371
	2	0.03 ± 0.03	0.286	0.04 ± 0.03	0.207	−0.02 ± 0.03	0.567

Abbreviations: APOE4: apolipoprotein E4; IGF-1: insulin-like growth factor-1; VEGF: vascular endothelial growth factor; BDNF: brain-derived neurotrophic factors; SE: standard error; DM: diabetes mellitus. Model 1: adjusted for age and sex. Model 2: additionally adjusted for body mass index, smoking, total cholesterol, triglycerides, lipid medication, and APOE4. Ambulatory activity = number of flights of stairs and city blocks walked each day, which were added together to quantify the total amount of ambulatory (walking) physical activity. Ambulatory activity was natural log transformed, sedentary time was square root transformed, and total physical activity (PAI) was inverse transformed, but we reversed the sign for total physical activity to make higher values consistent with more physical activity. VEGF was also natural log transformed. All variables were standardized to a mean of 0 and SD of 1 to facilitate comparison.

## Data Availability

The data supporting the results of this study have been deposited in dbGaP.

## Supplementary Materials

Supplementary Materials Supplemental Table 1: interactions by DM status in the relation of physical activity to IGF-1, VEGF, and BDNF. In this table, the study population was stratified by age group (< or ≥60 years). We present interactions for the relation of physical activity to IGF-1, VEGF, and BDNF by DM status in each stratum and in the total study population. Supplemental Table 2: relations of physical activity variables to IGF-1 and BDNF, stratified by age and DM status. In this table, the study population was stratified by DM status, then participants with or without DM were further stratified by age (< or ≥60 years). Relations of ambulatory activity to IGF-1 levels and total physical activity to BDNF levels were explored in these strata. Only those analyses were explored because we observed significant relations between only those variables in the main study results (among participants with DM). Supplemental Table 3: relations of age and DM status to circulating BDNF, IGF-1, and VEGF levels. In this table, the study population was stratified by DM status, then participants with or without DM were further stratified by age (< or ≥60 years). The mean (±SD) or median (Q1, Q3) for IGF-1, VEGF, and BDNF levels was presented for each strata.
