# Supplementary material for: Self-Reported Physical Activity and Relations to Growth and Neurotrophic Factors in Diabetes Mellitus: The Framingham Offspring Study
Source: J Diabetes Res. 2019 Jan 9;2019:2718465. doi: 10.1155/2019/2718465 (PMC6343169; doi:10.1155/2019/2718465)
Supplement: Supplementary Materials — Supplemental Table 1: interactions by DM status in the relation of physical activity to IGF-1, VEGF, and BDNF. In this table, the study population was stratified by age group (< or ≥60 years). We present interactions for the relation of physical activity to IGF-1, VEGF, and BDNF by DM status in each stratum and in the total study population. Supplemental Table 2: relations of physical activity variables to IGF-1 and BDNF, stratified by age and DM status. In this table, the study population was stratified by DM status, then participants with or without DM were further stratified by age (< or ≥60 years). Relations of ambulatory activity to IGF-1 levels and total physical activity to BDNF levels were explored in these strata. Only those analyses were explored because we observed significant relations between only those variables in the main study results (among participants with DM). Supplemental Table 3: relations of age and DM status to circulating BDNF, IGF-1, and VEGF levels. In this table, the study population was stratified by DM status, then participants with or without DM were further stratified by age (< or ≥60 years). The mean (±SD) or median (Q1, Q3) for IGF-1, VEGF, and BDNF levels was presented for each strata. [file 2718465.f1.docx]

**Supplemental Table 1.** Interactions by DM status in the relation of physical activity to IGF-1, VEGF and BDNF

|  |  | **Total Sample (n=1730)** | | | **<60 years (n=870)** | | | **≥60 years (n=860)** | | |
| --- | --- | --- | --- | --- | --- | --- | --- | --- | --- | --- |
|  |  | **IGF-1** | **VEGF** | **BDNF** | **IGF-1** | **VEGF** | **BDNF** | **IGF-1** | **VEGF** | **BDNF** |
| **Interaction by DM status** | **Model** | **p** | **p** | **p** | **p** | **p** | **p** | **p** | **p** | **p** |
| **Sedentary Time** | **1** | 0.473 | **0.024** | 0.610 | 0.146 | **0.077** | 0.930 | 0.490 | 0.164 | 0.375 |
|  | **2** | 0.687 | **0.027** | 0.518 | 0.244 | **0.099** | 0.748 | 0.379 | 0.144 | 0.405 |
| **Total Physical Activity** | 1 | 0.627 | 0.428 | **0.076** | 0.144 | 0.732 | 0.169 | **0.039** | 0.532 | 0.163 |
|  | **2** | 0.435 | 0.410 | **0.031** | 0.254 | 0.791 | **0.077** | **0.025** | 0.486 | 0.103 |
| **Ambulatory Activity** | **1** | **0.081** | 0.170 | 0.631 | 0.762 | 0.540 | 0.974 | **0.040** | 0.199 | 0.421 |
|  | **2** | 0.104 | 0.157 | 0.685 | 0.968 | 0.633 | 0.810 | **0.032** | 0.174 | 0.371 |

Abbreviations: Insulin-like growth factors (IGF)-1; vascular endothelial growth factor (VEGF); brain-derived neurotrophic factor (BDNF); diabetes mellitus (DM).

Model 1: adjusted for age and sex

Model 2: additionally adjusted for body mass index, smoking, total cholesterol, triglycerides, lipid medication, and APOE4

Ambulatory activity= number of flights of stairs and city blocks walked each day, which were added together to quantify the total amount of ambulatory (walking) physical activity. Ambulatory activity was natural log-transformed, sedentary time was square-root transformed, and total physical activity (PAI) was inverse transformed, but we reversed the sign for total physical activity to make higher values consistent with more physical activity. VEGF was also natural log-transformed. All variables were standardized to a mean of 0 and SD of 1 to facilitate comparison. Significant p-values for the interaction (p<0.1) were bolded for emphasis.

**Supplemental Table 2.** Relations of physical activity variables to IGF-1 and BDNF, stratified by age and DM status

|  |  | **Participants Without DM** | | | | **Participants With DM** | | | |
| --- | --- | --- | --- | --- | --- | --- | --- | --- | --- |
|  | **Model** | **<60 years**  **N=811** | **p** | **≥60 years**  **N=740** | **p** | **<60 years**  **N=59** | **p** | **≥60 years**  **N=120** | **p** |
| **Ambulatory activity to IGF-1,** | 1 | **0.09 ± 0.04** | **0.015** | -0.004 ± 0.04 | 0.915 | 0.12 ± 0.12 | 0.310 | **0.23 ± 0.10** | **0.028** |
|  | 2 | 0.08 ± 0.04 | 0.058 | -0.01 ± 0.04 | 0.751 | 0.06 ± 0.13 | 0.645 | **0.23 ± 0.11** | **0.042** |
| **Total physical activity to BDNF** | 1 | 0.02 ± 0.03 | 0.643 | -0.002 ± 0.04 | 0.952 | 0.20 ± 0.14 | 0.144 | 0.13 ± 0.10 | 0.214 |
|  | 2 | 0.007 ± 0.03 | 0.838 | -0.0002 ± 0.04 | 0.996 | 0.28 ± 0.15 | 0.073 | 0.18 ±0.10 | 0.074 |

Abbreviations: Insulin-like growth factors (IGF)-1; brain-derived neurotrophic factor (BDNF); diabetes mellitus (DM).

Model 1: adjusted for age and sex

Model 2: additionally adjusted for body mass index, smoking, total cholesterol, triglycerides, lipid medication, and APOE4

Ambulatory activity= number of flights of stairs and city blocks walked each day, which were added together to quantify the total amount of ambulatory (walking) physical activity. Ambulatory activity was natural log-transformed, sedentary time was square-root transformed, and total physical activity (PAI) was inverse transformed, but we reversed the sign for total physical activity to make higher values consistent with more physical activity. VEGF was also natural log-transformed. All variables were standardized to a mean of 0 and SD of 1 to facilitate comparison. Significant p-values (p<0.05) were bolded for emphasis.

**Supplemental Table 3.** Relations of age and DM status to circulating BDNF, IGF-1, and VEGF levels.

|  | **Participants Without DM** | | | **Participants With DM** | | |
| --- | --- | --- | --- | --- | --- | --- |
|  | **<60 years**  **N=811** | **≥60 years**  **N=740** | **p** | **<60 years**  **N=59** | **≥60 years**  **N=120** | **p** |
| **IGF-1, ng/ml**  mean ± SE | 126 ± 35 | 114 ± 35 | **<0.0001** | 117 ± 33 | 107 ± 39 | 0.10 |
| **VEGF, pg/ml**  median (Q1, Q3) | 277 (152, 414) | 276 (151, 439) | 0.67 | 241 (167, 431) | 284 (144, 475) | 0.98 |
| **BDNF, pg/ml**  mean ± SE | 24016 ± 7835 | 23729 ± 8446 | 0.49 | 25489 ± 8904 | 23705 ± 9588 | 0.23 |

Abbreviations: Insulin-like growth factors (IGF)-1; vascular endothelial growth factor (VEGF); brain-derived neurotrophic factor (BDNF); diabetes mellitus (DM); standard deviation (SD); quartile (Q).

P-values were calculated using a 2-sample t-test. For variables not normally distributed a transformation was applied to make the distribution more normal.

Significant p-values (p<0.05) were bolded for emphasis.
